# Microwave Synthesis of Molybdenum Disulfide Nanoparticles Using Response Surface Methodology for Tribological Application

**DOI:** 10.3390/nano12193369

**Published:** 2022-09-27

**Authors:** Thachnatharen Nagarajan, Mohammad Khalid, Nanthini Sridewi, Priyanka Jagadish, Rashmi Walvekar

**Affiliations:** 1 Faculty of Defense Science and Technology, National Defense University of Malaysia, Kuala Lumpur 57000, Malaysia; 2 Graphene and Advanced 2D Materials Research Group (GAMRG), School of Engineering and Technology, Sunway University, Subang Jaya 47500, Malaysia; 3 Sunway Materials Smart Science & Engineering (SMS2E) Research Cluster, School of Engineering and Technology, Sunway University, Subang Jaya 47500, Malaysia; 4 Department of Chemical Engineering, School of Energy and Chemical Engineering, Xiamen University Malaysia, Jalan Sunsuria, Bandar Sunsuria, Sepang 43900, Malaysia

**Keywords:** microwave synthesis, optimization, MoS_2_ nanoparticles, nanolubricants, tribology

## Abstract

We used response surface methodology (RSM) based on the central composite design (CCD) model to optimize the synthesis time and temperature of the molybdenum disulfide (MoS_2_) nanoparticles using the flexiWAVE microwave. Furthermore, the synthesized MoS_2_ nanoparticles were used in SAE 20W50 diesel engine oil to study the tribological properties according to ASTM standards using a four-ball tribotester. The optimization result shows that the synthesis temperature and time for the MoS_2_ nanoparticles in the microwave were ~200 °C and ~15 min, respectively, with a coefficient of friction (COF) and average wear scar diameter (WSD) of 0.0849 and 320 μm. Furthermore, the difference between the experimental and predicted values was minimal (1.88% (COF) and 0.625% (WSD)), which was similar to the optimization model.

## 1. Introduction

An enormous amount of energy is used to overcome the friction of moving objects. As a result, friction-related wear and heat can cause damage to the contact surface, material fatigue, unnecessary mechanical energy losses, noise emissions, and degraded machine efficiency [1]. Friction and wear are two fundamental causes of the breakdown of engineering parts in various structures, such as gears and valves. The price of machinery, fitting, and maintenance due to frictional defects, wear, and tear put immense burdens on the nation’s economy. Approximately a third of fuel is utilized in passenger vehicles to subdue friction in engines, transmissions, and braking [2]. A decrease in energy usage can be accomplished mainly by enhancing the tribological properties of system surfaces. The specifications for improved lubricants are increasingly challenging due to the usability of their properties across a broader temperature range, higher loads, higher speed, improved reliability, and service life.

Military armored vehicles with diesel-based engines experience massive heat generation and pressure due to extensive driving in uneven terrains with bulky equipment. In order to ensure the mechanical parts are working efficiently and to increase the service life of the vehicle’s engine, diesel-based engine oil must manage friction effectively and minimize wear for the engine’s mechanical components [3].

The anti-friction additive is critical in the tribology of diesel-based engine oil, especially for military vehicles with rapidly evolving mechanical equipment. As a result, the load on a heavy-duty vehicle engine per unit mass increases, making it difficult for traditional lubricant additives to meet the demands of extreme operating conditions in modern diesel engine components [4,5]. Therefore, developing new and effective friction-resistant plus high-bearing lubricant additives is critical to meet the demands of powerful machinery in extreme working conditions.

One of the leading scientific challenges is producing new lubricants that satisfy the evolving criteria in various strategic fields such as transportation, manufacturing, and defense. In recent years, researchers have established that nanotechnology can be the most innovative aspect of science in the twenty-first century [6]. Continuous advances in science and technology provide an outstanding forum for nanotechnology to evolve at a faster pace. As a result of development, researchers have also discovered that the tribological properties of lubricants could be improved by including nanoparticles, which would significantly decrease the coefficient of kinetic friction in operating devices [7,8].

Several nanoparticles consist of two adjacent layered structures, bound by weak van der Waals forces, responsible for lowering the shear strength and causing sliding or lubricating effects on the system’s active adjacent layer structure [9,10]. Furthermore, two-dimensional (2D) nanomaterials have a larger specific surface area than other nanomaterial surfaces, allowing them to cover a large surface area during absorption on a substrate exterior, removing the kinetic friction between two contact surfaces [11].

Due to its physical and chemical stability in lubrication, molybdenum disulfide (MoS_2_) is currently regarded as a high-potential 2D transition metal chalcogenide. The material is chemically balanced, resistive to most acids, and immune to irradiation. It is both a semiconductor and diamagnetic in its purest form. The lubricant rate depends on its crystalline lamella structure, where the sulfur lamellae are linked by a weak van der Waals interaction, reducing the fiction [12]. During sliding, the crystalline layers of MoS_2_ would effectively slide and align parallel to the relative movement, which causes the lubrication effect. However, the powerful ionic bond between S and Mo makes the lamellar highly resistant to the penetration of asperities [13]. Nanostructure research has also been on the rise in the last few years. For example, using MoS_2_ nanocrystals for lubrication will produce a superlubricity framework (a coefficient of friction lower than 0.01) [14]. Several theories have been proposed for this phenomenon, which has also been observed in fullerene configurations and nanotubes, where nanostructures act as nano bearings in tribological contact, lowering the COF of the mechanism significantly [15,16]. For the synthesis of MoS_2_ nanoparticles, various preparatory methodologies have been established, including high-temperature sulfurization, thermal reduction, hydrothermal process, laser ablation, and even chemical vapor deposition (CVD) [17,18,19,20]. However, the advanced microwave synthesis of MoS_2_ nanoparticles has rarely been documented, and its use in the tribology field has not been published in the literature. 

Hydrothermal and microwave synthesis techniques have been employed to synthesize MoS_2_ nanoparticles at comparatively larger yields. The hydrothermal method is frequently used due to the accessibility of the processing equipment, but it suffers from a lack of even heating. However, substances can also be heated rapidly in the microwave synthesis process, producing a consistent temperature ramp relative to traditional oven-based hydrothermal processes. Furthermore, the reaction Teflon vessels are translucent to the microwave and will ensure continuous heating throughout the reaction vessels. In addition, the microwave gains from rapid and accelerated heating, high-temperature homogeneity, and selective heating over traditional methods [21]. The reactions primarily depend on their precursors’ ability, including solvents to consume microwave energy efficiently. The above findings confirm that the microwave synthesis technique is superior to the hydrothermal technique due to its uniform heating, low energy consumption, higher yield, and shorter synthesis. In some papers [22,23,24], traditional heating in the oven that takes approximately 24 h is employed to synthesize the MoS_2_ nanosheets, whereas microwave synthesis takes less than 30 min.

The novelty of this experiment is to investigate the optimization of the microwave-assisted synthesis of MoS_2_ nanoparticles for tribological application using a response surface methodology (RSM) approach with a central composite design (CCD) model under Design Expert (Stat-Ease). Most previous studies were carried out using a univariate approach where only one element is varied at a time, often resulting in missing experimental data. However, with RSM and the CCD model, this optimization approach investigates a larger experimental domain [25]. Furthermore, the two vital experimental parameters for synthesis, such as temperature and time vary together, resulting in higher optimum values. Therefore, the principal purpose of this study was to identify the optimum time and temperature needed to synthesize the MoS_2_ via microwave that gives the best tribological results in military-grade diesel-based engine oil. Overall, this research highlights the effects of microwave synthesized nanoparticles on the tribological criteria of engine oil.

## 2. Materials and Methods

### 2.1. Materials

All of the chemical substances used in the investigation were of analytical grade and were not further purified. The chemicals used for the preparation of MoS_2__,_ such as ammonium molybdate tetrahydrate ((NH_4_)_6_Mo_7_O_24_·4H_2_O) and thiourea (SC(NH_2_)_2_), were purchased from Fisher Scientific Leicester, England, UK, and R&M Chemicals Petaling Jaya, Selangor, Malaysia. The base oil used was the SAE 20W50 diesel engine oil.

### 2.2. Preparation of MoS_2_ Nanoparticles Using Microwave

All chemical reagents were measured using an analytical balance with the precision of ±0.1 mg (Mettler Toledo, Greifensee, Switzerland). A total of 1 mmol of ammonium molybdate tetrahydrate ((NH_4_)_6_Mo_7_O_24_·4H_2_O) and 30 mmol thiourea (SC(NH_2_)_2_) were dissolved in 35 mL of deionized water. First, the solution was stirred for 20 min at room temperature. Then, the obtained homogeneous solution was transferred into a microwave advanced flexible microwave synthesis platform (flexiWAVE Milestone, Sorisole, Italy) Teflon vessel. Twelve different samples of MoS_2_ were synthesized according to the time and temperature combinations generated by the Design-Expert, version 9, Software For Statistical Computation, Stat-Ease software, Minneapolis, MN, USA, 2019, as shown in Table 1. After the reaction mixtures had cooled to room temperature, the samples were centrifuged and washed with deionized water and ethanol multiple times and then dried in a vacuum oven at 70 °C for 12 h.

### 2.3. Experimental Design and Statistical Analysis

Theoretic assumptions based on scientific findings are the core of the beneficial analysis. There are two fundamental areas of interest in scientific experimentation: design of the experiment and the statistical analysis of the results. The design of experiments (DOE) aims to evaluate the important parameters for understanding variance in the process [26]. DOE also tries to consider how influential forces are interfering with the system.

The response surface method (RSM) was used in this study to examine the effect of input parameters on the response parameters. RSM is a set of mathematical and computational approaches that can be used to describe and evaluate problems in which multiple variables influence the solution of interest. For example, if all input parameters depict quantitative variables, the response could be interpreted as functional stages and variables, as shown by Equation (1).

Y = f (X_1u_, X_2u_, …, X_iu_) + E_u_ (1)

where u = 1, 2, …, N represents N observations in the empirical studies, and Xiu shows the degree of ith factor of uth observation. Function f is considered the function of response. The residual Eu measures the experimental error of the u^th^ measurements.

The RSM algorithm employs a factorial design, with the main effects defined as the difference in response caused by a change in the reasoned factor while all other factors remain constant. A polynomial regression modifies the experimental results to the above equation, and the standard statistics can be used to determine the model’s fitness. The analysis of variance (ANOVA) must be conducted to assess the significance of the established model and the importance of the specific coefficient model. The ANOVA describes the critical consequences and relationships, the regression coefficients, and the *p*-value. The F-value and *p*-value of the ANOVA study facilitate assessing the results, which is that the factors and interactions are statistically important. The lower the *p*-value, the lower the probability of an error by declining the null hypothesis. It is also proposed that the *p*-value be less than 0.05, making the model meaningful at the 95% confidence level. The ANOVA was also performed to explain the validity and adequacy of the regression model. In order to determine the fitness of the experiment, the value of the correlation coefficient (R^2^) was used, and the statistical significance of the model equation was tested using the F test.

This research used RSM to optimize two experimental parameters: (1) the synthesis temperature and (2) synthesis time required for the microwave synthesis of MoS_2_ on the tribological activity of nanolubricants tested using a four-ball tribotester. The friction coefficient and specific wear rate were used as response factors. The program Design-Expert version 9 (Stat-Ease) was used for research, and the tests were formulated using the central composite design (CCD) model. Based on the CCD configuration, 12 experimental runs with different times and temperatures synthesized MoS_2_ nanoparticles using the microwave were generated.

### 2.4. Formulation of the Nanolubricant

The constant 0.05 wt.% of the obtained MoS_2_ nanoparticles were dispersed in 100 mL of SAE 20W50 military-grade diesel engine oil with the help of a homogenizer at 7000 rpm for 10 min. The samples were sonicated using a sonication bath for 30 min to ensure that the nanoparticles were uniformly dispersed in the base oil. The formulated nanolubricants showed high stability for more than a week.

### 2.5. Tribological Study

**Figure 1:**
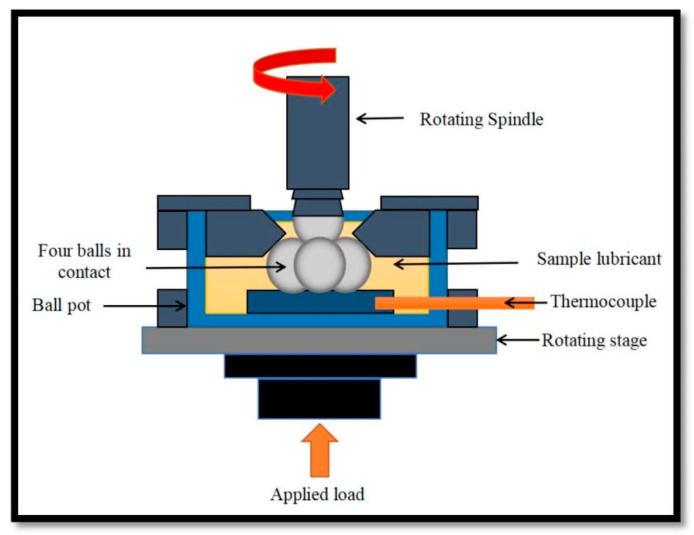
A schematic drawing of the experimental configuration of the four-ball tribotesters.

The coefficient of friction (COF) and wear scar diameter (WSD) of the nanolubricant were examined by a four-ball tribotester (DUCOM). Tribological tests were performed on steel balls submerged in nanolubricants, with the upper ball rotating against the lower three balls held in a fixed position in the ball pot. The metal ball bearings employed in the tests were equivalent to 12.7 mm in diameter, and the mechanical properties of the metal ball bearings utilized are presented in Table 2. Before the experiment, the steel balls and other equipment were cleaned with ethanol and dried to deter impurities. All testing parameters, including rotating speed, applied load, time, and temperature, were 12,000 rpm, 392.5 N, 3600 s, and 75 °C, respectively, as per the ASTM standards. Figure 1 shows the schematic drawing of the experimental configuration of the four-ball tribotesters. The main data processor connected to the tribotester recorded the nanolubricant COF, and the diameter of the wear scar was measured using image acquisition devices.

### 2.6. Characterization of Nanoparticles

The characterization of the MoS_2_ nanoparticles for the particle morphology and size distributions was confirmed using a field emission scanning electron microscope (FESEM, HITACHI SU6600 Dublin, Ireland) and energy dispersive spectroscopy (EDS, HORIBA-EMAX Dublin, Ireland) for the nanoparticle composition. In addition, an X-ray diffractometer was used to collect the XRD data. The samples were scanned from 20 to 80 degrees at a step size of 1 degree/min with a slit divergence of 0.9570 degrees. The analysis was conducted using copper K-alpha radiation of wavelength 1.54 angstroms, and X-rays were filtered through Ni using an operational voltage of 45 kV and a current of 27 mA.

## 3. Result and Discussion

### 3.1. Design of Experiments and Analysis of Variance (ANOVA)

The CCD model with two experimental factors (synthesis temperature and time) was used to determine the outcome of these experimental components on the COF and average WSD of the nanolubricant. The design of experiments generated by the CCD model with varying synthesis conditions and the experimental values of COF and the average WSD of the nanolubricant are shown in Table 3. The following evaluation was carried out to reach the precision of the model: ANOVA analysis, normality assessment regression analysis, and residual analysis for the COF and average WSD. After adequate completion of the above experiments in the range of the required statistical limits, the model equation was established. 

#### 3.1.1. Effect of Microwave Synthesis Temperature and Time on COF

Table 4 displays the ANOVA study of the COF produced by the nanolubricant with the microwave synthesized MoS_2_ nanoparticles. In the current study, the confidence level of the CCD model was maintained at 95%. The F value of the model for the MoS_2_ nanolubricants was 55.80, and a *p*-value < 0.0001 indicated that the applied model was significant with a marginal noise effect on the COD of the nanolubricants. The lack of fit of the F and *p* values was not significant, indicating that the chosen CCD model fit well with the COF in the experimental dataset.

Careful analysis of the F and *p* values revealed that factor B (time) had a more significant effect on the COF of the nanolubricants of MoS_2_ than factor A (temperature). The F test also projected the importance of factor B (time) to the nanolubricants. The statistical accuracy was also tested to ensure the model’s predictive capacity, as seen in Table 5. The approximate R^2^ values for the COF for the nanolubricants were 0.9789, which suggests an adequate description of the real interaction between the different experimental variables for the model. Good precision, a calculation of the signal-to-noise ratio, was observed to be 21.926 for the nanolubricants, as seen in Table 5. These values confirmed the precision of the formula was higher, as the ratio was higher than 4. This model can also be deployed to traverse the design space.

**Figure 2:**
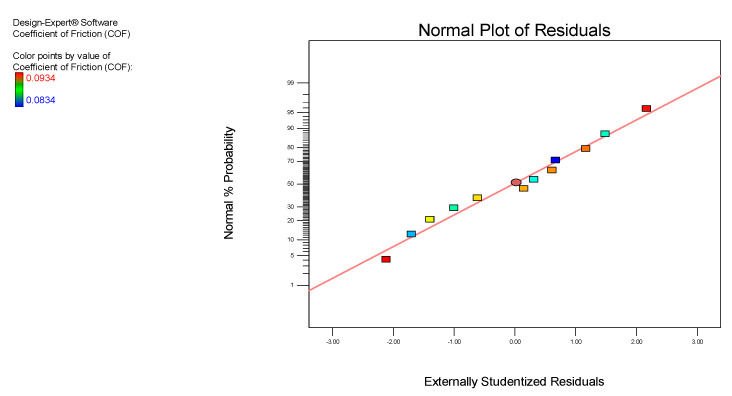
The normal probability plot of the COF for the MoS_2_ nanolubricants.

Figure 2 displays the standard probability graph of the COF for the nanolubricants of MoS_2_. The standard probability graph examines the experimental normality outcomes and displays the predicted versus actual values for the configuration matrix. For the ANOVA analysis, the standard probability graph must be tested for the residual range that should be closest to the mean line. Figure 2 shows that the residual values were minimal and closely associated with the mean line displayed in the graph. 

The experimental outcome of the COF for the MoS_2_ nanolubricants was fitted to a quadratic polynomial equation, as shown in Equations (2) and (3).

Coefficient of Friction (COF) = (0.092) + (−1.199 × 10^−4^ × A) + (−2.781 × 10^−3^ × B) + (−1.920 × 10^−3^ × AB) + (−2.805 × 10^−3^ × A^2^) + (−8.550 × 10^−4^ × B^2^) (2)

Coefficient of Friction (COF) = (−0.37880) + (4.86057 × 10^−^^3^ × A) + (+4.86339 × 10^−^^3^ × B) + (−2.55975 × 10^−^^5^ × AB) + (−1.24665 × 10^−^^5^ × AB) + (−1.24665 × 10^−^^5^ × A^2^) + (−3.41981 × 10^−^^5^ × B^2^) (3)

where A = temperature (°C), B = time (minutes).

**Figure 3:**
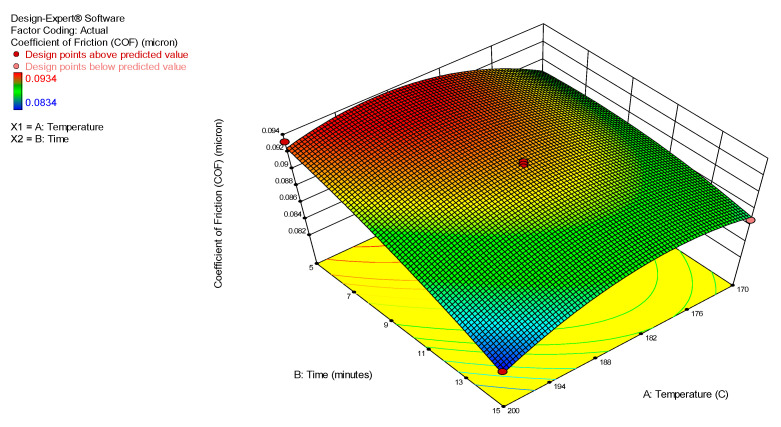
A 3D interaction plot of the COF for the MoS_2_ nanolubricant.

**Figure 4:**
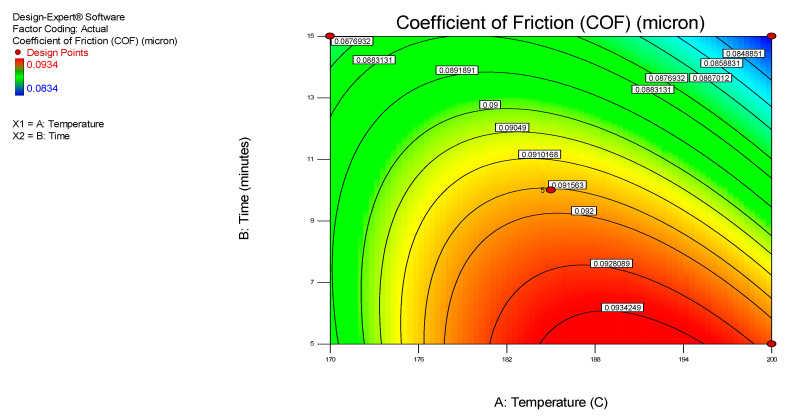
A contour interaction plot of the COF for the MoS_2_ nanolubricants.

Figure 3 and Figure 4 display the 3D surface response and contour plots, representing the regression equation acquired from the developed model. This was utilized to analyze the relationship between the experimental parameters, such as the synthesis temperature and time and its corresponding optimum values, to achieve the lowest COF using the nanolubricant MoS_2_. In addition, the elliptical or saddle form of the contour plot determines the value of the relationship, and an elliptical or saddle plot can be achieved where there is ideal interaction with the independent variables [27]. Moreover, Figure 3 and Figure 4 graphically illustrate the relationship between the synthesis temperature and time on the COF for the MoS_2_ nanolubricants. Both plots clearly show that as the time and temperature variables of the MoS_2_ microwave synthesis increased, the COF of the nanolubricant decreased. The dark blue area represents the lowest COF of the nanolubricant. The dark blue area represents a large region with the lowest frictional values (<0.08) at the time above 15 min and a temperature around 200 °C. The crystallinity of the MoS_2_ nanoparticles improved as the microwave synthesis time and temperature increased. The crystallinity of the nanoparticles was attributed to their mechanical strength and improved tribological properties by reducing the COF of the nanolubricant-based MoS_2_. Residual analysis was carried out due to the close approximation of the actual system. Residuals (*ri*) were extracted from the following regression in Equation (4):

*ri* = *yi*
*observed* − *yi*
*predicted* (4)

where *r* is the residuals, *y* is the response, and *i* is the observation.

**Figure 5:**
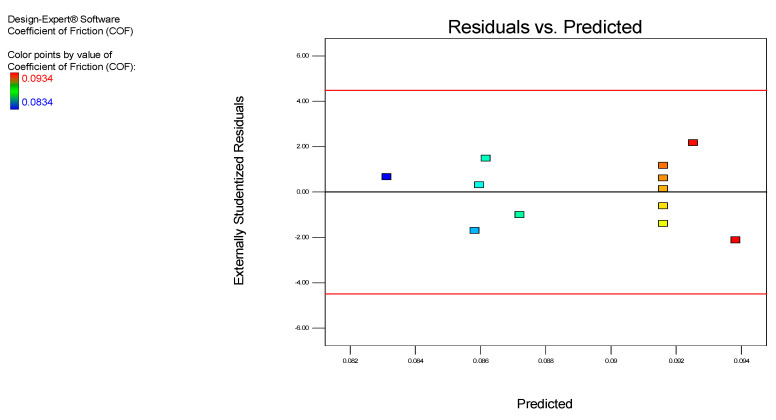
The residuals vs. predicted COF for the MoS_2_ nanolubricants.

**Figure 6:**
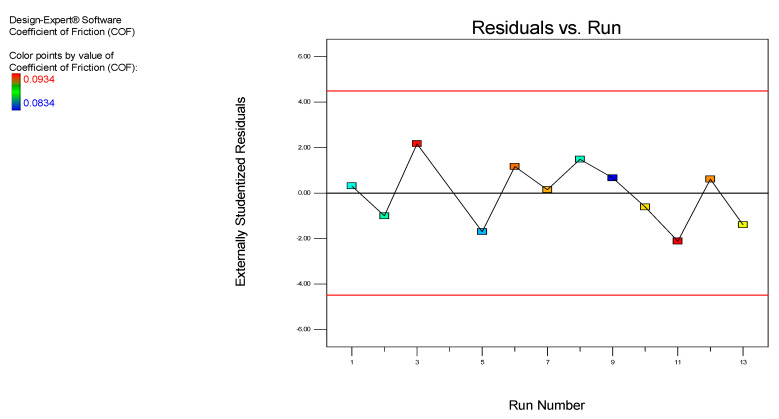
The residuals vs. the experimental run of the COF for the MoS_2_ nanolubricants.

The value of all the residual observations used in the residual plot involves the residual vs. the predicted plot and the residual vs. the experimental run plot. The residual vs. predicted plot and the residual vs. experimental run plot of the COF for the MoS_2_ nanolubricants are shown in Figure 5 and Figure 6, respectively, which is the significant diagnosis for the model. According to Draper and Smith [28,29], linear relationships are normal in error terms. Our results have shown no defects, suggesting that errors obey the normal distribution and endorse the experimental model.

**Figure 7:**
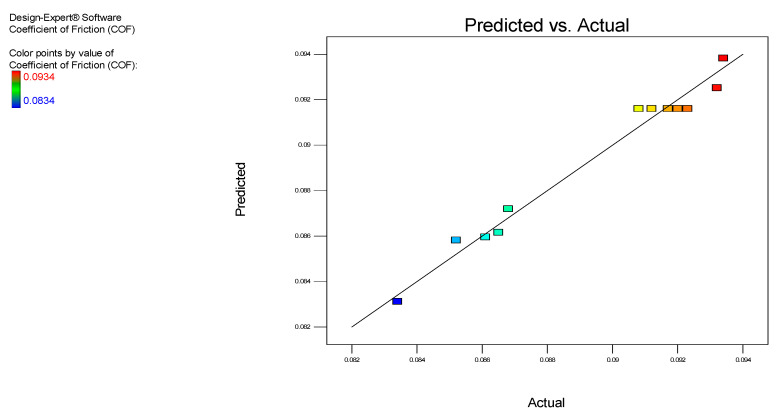
The predicted vs. the actual COF for the MoS_2_ nanolubricants.

In addition, an irregular pattern of scattering was observed from the residual vs. the predicted plot in Figure 5. The residuals were well-proportioned in positive and negative residues within a gradient of −2 ˂ *ri* ˂ +2 (*ri* is actual residuals). Moreover, in Figure 6, no trend matched the residual vs. the experimental run plot, which confirms that not all residues are associated with one another due to time-related variables. The established model is appropriate, with no indication of any violation of the objectivity or the constant variance hypothesis. Figure 7 displays the predicted vs. the actual COF results for the MoS_2_ nanolubricants. The points were irregularly scattered along the 45-degree line and indicated the accuracy of the predicted data on the actual data. It remarks on the design, and the results validate the excellent predictability.

#### 3.1.2. ANOVA Analysis of Average WSD

The contact area’s wear rate in the thin film lubrication regime is a critical parameter in tribological experiments, along with the COF used to select the required lubricant. Therefore, the WSD investigation is considered as one of the conventional methods of recognizing the wear output of lubricating oil. The wear scars were created due to the spindle’s sliding motion in a four-ball machine, and an image acquisition system was utilized to analyze and scale the WSD of each ball. Additionally, the average WSD of the fixed balls was determined using Equation (5).

*AverageWSD* (*μm*) = [*scar* (1) *diameter* + *scar* (2) *diameter* + *scar* (3) *diameter*]/3 (5)

**Figure 8:**
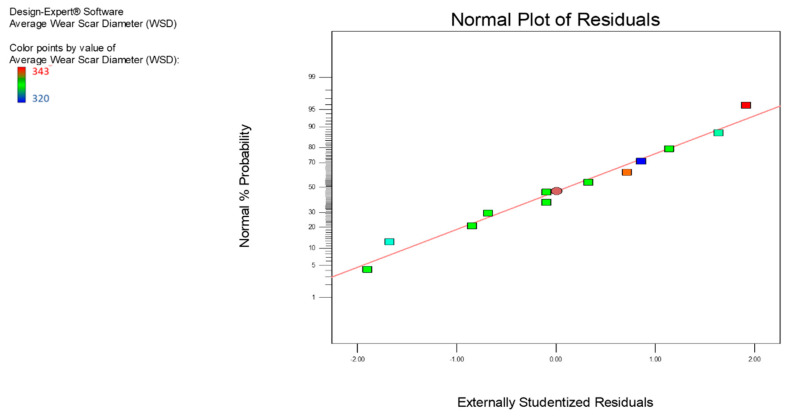
The normal probability plot of the average WSD for the MoS_2_ nanolubricants.

ANOVA study of the average WSD was performed using the same design methods to analyze the COF. The overview of the ANOVA analyses for nanolubricants can be seen in Table 6 for the association of process parameters and the F and *p* values. The temperature is a less critical parameter within the chosen confidence degree, while the time is a more significant parameter for nanolubricants when referring to the F value. According to Table 7, the approximate value of R^2^ for the model developed for a particular rate of wear for the MoS_2_ nanolubricants was 0.9474, which is satisfactory. The modified R^2^ values of 0.9174 were similar to the respective R^2^ values that confirmed the model’s fair predictability within the parametric range domain. The regression equations obtained from the model and evaluated for normality (Figure 8) are given in Equations (6) and (7), respectively, for the nanolubricants of MoS_2_. The data plotted in Figure 7 exhibited good behavior, as the residual data were very minute and closely associated with the mean line. Thus, the data showed a good agreement with the model.

Average Wear Scar Diameter (WSD) = (+334) + (−5.095 × 10^−4^ × A) + (−2.022 × 10^−3^ × B) + (−2.580 × 10^−3^ × AB) + (−5.390 × 10^−4^ × A^2^) (6)

Average Wear Scar Diameter (WSD) = (−0.047524) +(1.19638 × 10^−3^ × A) + (5.95843 × 10^−3^ × B) + (−3.43938 × 10^−5^ × AB) + (−2.39569 × 10^−5^ × A^2^) (7)

where A = temperature (°C), B = time (minutes).

**Figure 9:**
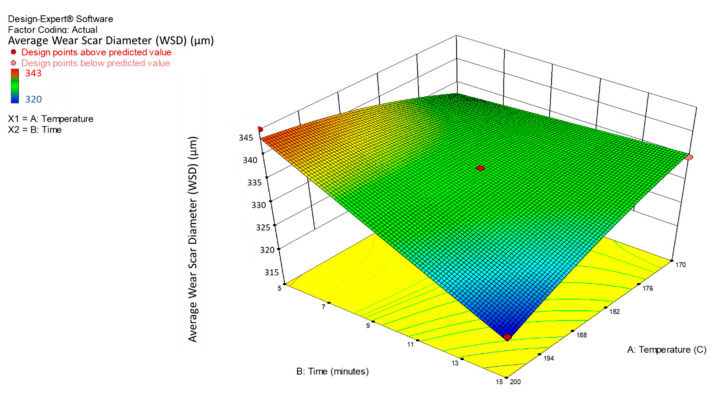
A 3D interaction plot of the average WSD for the MoS_2_ nanolubricants.

**Figure 10:**
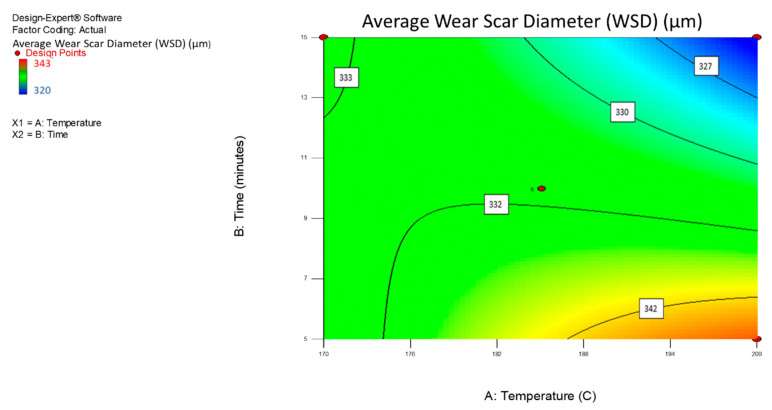
A contour interaction plot of the average WSD for the MoS_2_ nanolubricants.

The interaction between various times and temperatures on the average WSD for the MoS_2_ nanolubricants was examined and demonstrated using 3D response surface and contour plots. From the quadratic model mentioned earlier (Equations (6) and (7)), the surface response and contour charts showed the interaction effect of the average WSD of the MoS_2_ nanolubricants in Figure 9 and Figure 10. The required time and temperature selection for the advanced microwave synthesis of MoS_2_ are crucial in this analysis to determine the average WSD for nanolubricants. It is clear from Figure 9 and Figure 10 that the increase in the synthesis time and temperature contributed to a lower average WSD for the MoS_2_ nanolubricants.

It was found that when the MoS_2_ was synthesized at higher temperatures (~200 °C) and duration (~15 min), it resulted in the lowest average WSD of 320μm. This showed a linear relationship between the time and temperature and average WSD; as the time and temperature increase, the average WSD decreases, but above 15 min of synthesis time, the average WSD increases. Therefore, from the data shown above, it can be inferred that when the precursors of MoS_2_ are subjected to the optimum microwave synthesis time and temperature, the average WSD during tribological studies is decreased. This effect arises when well-formed MoS_2_ with higher crystallinity has a lower WSD due to the formation of tribofilm between the contact surface [30].

**Figure 11:**
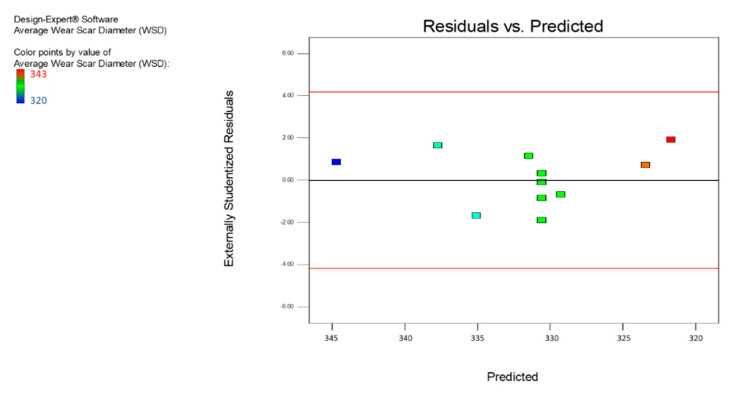
The residuals vs. the predicted average WSD for the MoS_2_ nanolubricants.

**Figure 12:**
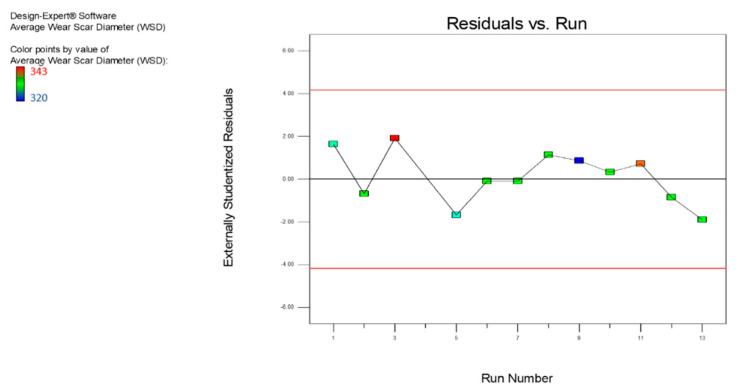
The residuals vs. the experimental run of the average WSD for the MoS_2_ nanolubricants.

**Figure 13:**
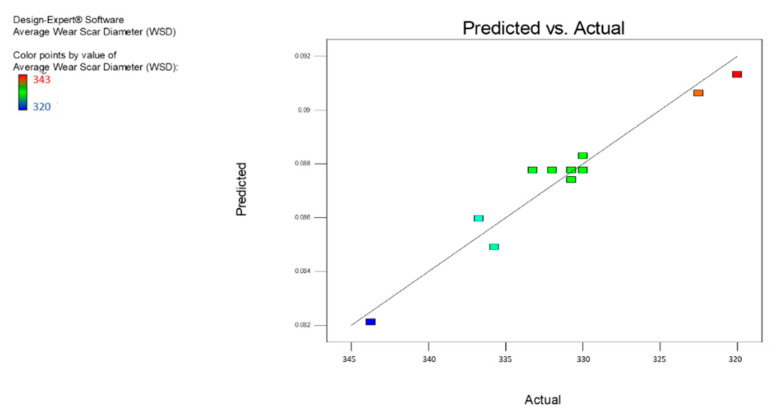
The residuals vs. the experimental run of the average WSD for the MoS_2_ nanolubricants.

Figure 11 represents a plot for the residual vs. the predicted values typically used to define or validate the presumption of constant variance. The graph showed a strong constant variance, and the values were well-spaced and randomly distributed along the line outcomes; therefore, the model correctly matched the variances. Figure 12 displays the residual versus run map, where the values were uniformly distributed, and most of the values were within the positive range. There were no outliers and extreme points in the chart, which means that the model fit strongly aligned with the run. Figure 13 provides a contrast between the expected and the actual values, showing that they strongly aligned with the response result (average WSD). The plot showed that more than 90% of the actual values fit the predicted values.

### 3.2. Characterization of MoS_2_

#### 3.2.1. Field Emission Scanning Electron Microscope (FESEM) and Energy Dispersive X-ray Spectroscopy (EDS) of Optimized MoS_2_ Nanoparticle

**Figure 14:**
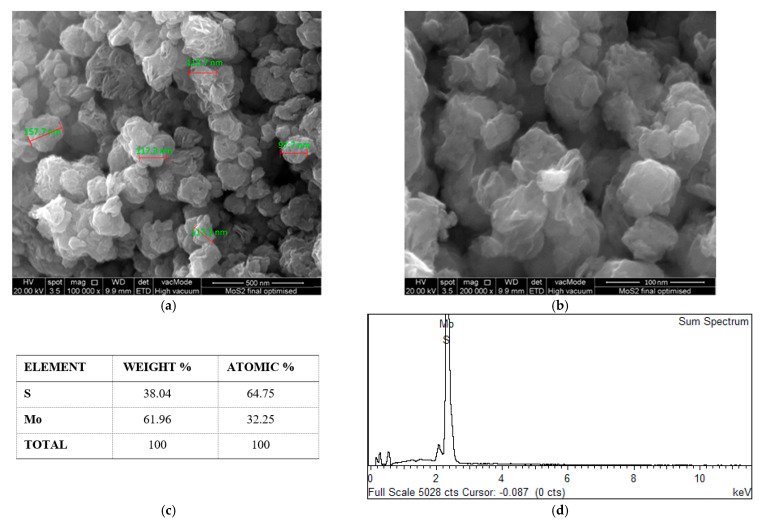
The MoS_2_ nanoparticles. (**a**,**b**) FESEM of the MoS_2_ nanoparticles at two magnification levels. (**c**) Composition of the MoS_2_ nanoparticles. (**d**) EDS spectrum of the MoS_2_ nanoparticles.

The FESEM images of the MoS_2_ nanoparticles confirmed the layered lamellar structure of the MoS_2_ nanoparticles at two different magnifications from Figure 14a,b. Furthermore, Figure 14c,d depicts the EDS analysis of the MoS_2_ nanoparticles based on their atomic and weight percentage, where the quantitative surface analysis of EDS performed in terms of the atomic and weight percentage of elements on the MoS_2_ nanoparticles revealed the existence of sulfur and molybdenum.

#### 3.2.2. X-ray Diffraction of Optimized MoS_2_ Nanoparticle

**Figure 15:**
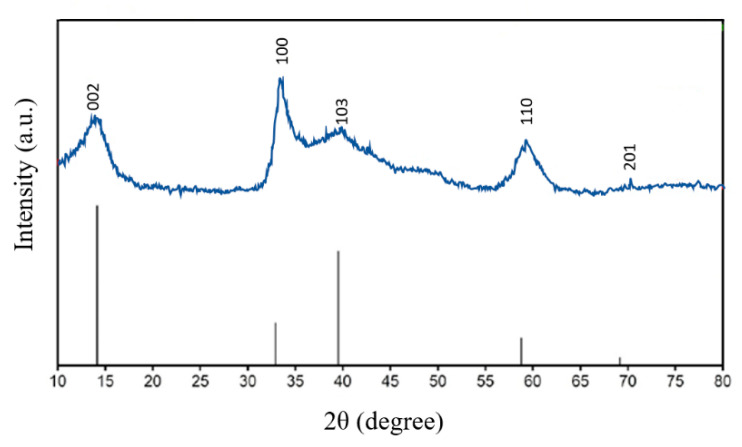
The XRD pattern of the MoS_2_ nanoparticles.

Figure 15 shows the XRD diffraction peaks of MoS_2_ at 2 = 14.5°, 33.0°, 39.3°, 58.5°, and 69.7°, which can be indexed as the (002), (100), (103), (110), and (201) peaks of the pure hexagonal MoS_2_ phase (JCPDS card no.371492), which are in accordance with previous studies [31,32]. Peak broadening can be seen, implying that the crystalline size is very small. For the (100) and (103) XRD peaks, the intensity variation between the reference pattern in the JCPD card and the synthesized sample was due to the differences in texture of the crystallite size difference and the size of the scattering domains. No other impurity peaks or separate phases existed in the XRD patterns, indicating that the crystal structure was made of pure MoS_2_ nanosheets. The crystallite size was estimated by using the Scherrer Equation (8):

(8) $$D=\frac{K\lambda }{\beta \cos\theta }$$

where *D* is the crystallite size (nm); *K* = 0.9 (Scherrer constant); *λ* is the wavelength of X-rays; *β* is the full width at half maximum (FWHM); and *θ* represents the peak position. According to Equation (8), the crystallite size of the MoS_2_ nanoparticles was 53.6 nm.

According to the characterization of the MoS_2_ nanoparticles, the nanolubricants improved the tribological properties due to adequate exfoliation force at the contacting surface and the configuration of tribofilms between the contact exterior. Furthermore, sufficient exfoliation pressure causes the deformation of nanoparticles required for the sliding effect, which promotes tribological properties. This exfoliation and deformation of nanoparticles result in the occupancy of MoS_2_ nanoparticles in the asperity contacts of the ball-bearing contact surfaces in the four-ball tribotester, resulting in the formation of the tribofilm [33]. This clearly shows that the laminar tribofilm is responsible for reducing the friction and anti-wear properties of nanolubricants rather than tribo-chemical reactions involving the MoS_2_ nanoparticles [34].

### 3.3. Optimization of Time and Temperature for MoS_2_ Microwave Synthesis for Tribological Application

**Figure 16:**
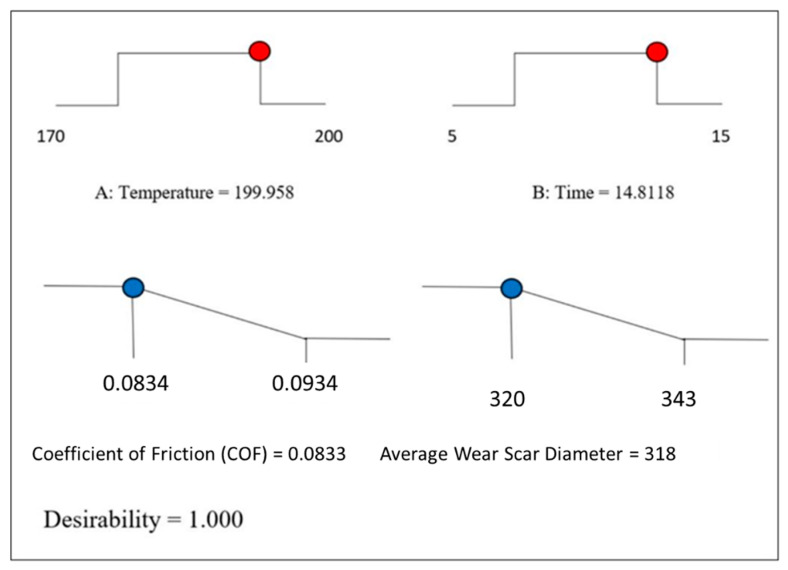
The ramp function plot for the optimization of the MoS_2_ nanolubricants.

The COF and average WSD are the two key characteristics of tribology, the interpretation of which is described in the preceding parts. Beyond the effectiveness of nanoparticle additives in lubricants, the synthesis approach with optimal temperature and time for reactions to favorable responses is necessary. As an outcome, multiple objective optimization methods have been developed and integrated into design of expert (DOE) software with the aid of desirability features. The optimized synthesizing time and temperature obtained from the DOE software were validated to verify the discrepancy in the expected and experimental values. The optimization procedure was performed at rotating speed, applied load, time, and temperature of 12,000 rpm, 392.5 N, 3600 s, and 75 °C, respectively, as per the ASTM standards. According to Figure 16, the optimum synthesis time and temperature of MoS_2_ through the microwave for the best tribological performance was ~200 °C and ~15 min with 1.000 desirabilities in the SAE 20W50 diesel engine oil. The predicted COF and average WSD were 0.0833 and 320μm, respectively.

With the optimized synthesis time and temperature of the MoS_2_ nanoparticles, real-time analyses were carried out to calculate the lowest COF and the average WSD of the nanolubricants. The model outcomes for the COF and average WSD were confirmed with in situ experimental results at the optimal synthesis time and temperature of the MoS_2_ nanoparticles and are shown in Table 8. As predicted, the experimental findings showed a reduction in friction and anti-wear characteristics (Table 8) with the inclusion of MoS_2_ nanoparticles. The experimental results for the COF and average WSD with the error percentage values were 0.0849 (1.88% error) and 320 (0.625% error), respectively, for the MoS_2_ nanolubricants. The error percentage values demonstrated a proximity prediction between the predicted and actual properties. These error values explicitly showed the model’s accuracy in relation to the domain of the experimental operating conditions. Table 9 shows the percentage enhancement of the COF (10.25%) and average WSD (10.60%) after the addition of the MoS_2_ nanoparticles in the base oil.

## 4. Conclusions

The lowest COF and WSD using time and temperature as parameters for synthesizing MoS_2_ nanoparticles were successfully achieved through design of expert (DOE) software. In addition, DOE analysis based on the response surface method (RSM) using the central composite design (CCD) and ANOVA has been proven to be a promising method to evaluate important parameters and maximize the operational factors related to the tribological properties of the MoS_2_ nanolubricants. Furthermore, the study of ANOVA, normality assessment, regression analysis, residual analysis, surface response plots, and contour plots demonstrated a close relationship between the experimental outcomes and the model’s predicted values.

The optimized temperature and time generated were ~200 °C and ~15 min, respectively, with 1.000 desirability conditions predicting a COF and an average WSD response of 0.0833 and 318 μm, respectively. The R-squared values of each analysis were 0.9789 (COF) and 0.9474 (WSD), which suggests a strong correlation with the model fit. The experimental effect of the COF and the average WSD for the optimized synthesizing time and temperature of the MoS_2_ nanoparticles was 0.0849 (COF) and 320 μm (WSD), consistent with the predicted values of 0.0833 and 318 μm, respectively. The discrepancy between the experimental and the predicted values was 1.88% (COF) and 0.625% (WSD), confirming the accurate prediction of the experimental parameters by DOE.

## Figures and Tables

**Table 1:** The parameter ranges selected for the study using CCD.

Coded Name	Variable Name	Type	Parameter Range	Parameter Unit
A	Temperature	Continuous	Level 1/low 170 Level 2/high 200	°C
B	Time	Continuous	Level 1/low 5 Level 2/high 15	Minutes

**Table 2:** The mechanical details of the metal ball bearing.

Properties	Ball Bearing
Material	Carbon-chromium steel
Hardness (*H*), HRC	1
Density (ρ), gm/cm^3^	7.79
Surface roughness (Ra), μm	0.022

**Table 3:** The experimental design and results.

	Factor 1	Factor 2	Response 1	Response 2
Run	Synthesis Temperature (°C)	Synthesis Time (Minutes)	Coefficient of Friction (COF)	Average Wear Scar Diameter (WSD) (µm)
1	185	17.0711	0.0861	331
2	170	15	0.0868	333
3	200	5	0.0932	343
4	206	10	0.0852	327
5	185	10	0.0923	334
6	185	10	0.0917	333
7	164	10	0.0865	334
8	200	15	0.0834	320
9	185	10	0.0912	333
10	185	3	0.0934	342
11	185	10	0.092	334
12	185	10	0.0908	333

**Table 4:** The ANOVA table for the COF of the MoS_2_ nanolubricants.

Source	Sum of Squares	Degrees of Freedom (df)	Mean Square	F-Value	*p*-Value Prob > F	Significance
Model	1.329 × 10^−4^	5	2.659 × 10^−5^	55.80	<0.0001	Significant
A-Temperature	8.626 × 10^−8^	1	8.626 × 10^−8^	0.18	0.6853	-
B-Time	4.639 × 10^−5^	1	4.639 × 10^−5^	97.35	<0.0001	-
AB	8.846 × 10^−6^	1	8.846 × 10^−6^	18.56	0.0050	-
A2	5.103 × 10^−5^	1	5.103 × 10^−5^	107.10	<0.0001	-
B2	4.741 × 10^−6^	1	4.741 × 10^−6^	9.95	0.0197	-
Residual	2.859 × 10^−6^	6	4.765 × 10^−7^	-	-	-
Lack of Fit	1.399 × 10^−6^	2	6.995 × 10^−7^	1.92	0.2608	Not significant
Pure Error	1.460 × 10^−6^	4	3.650 × 10^−7^	-	-	-
Cor Total	1.358 × 10^−4^	11	-	-	-	-

**Table 5:** The model summary of the quadratic model for the COF.

R-Squared	Adjusted R-Squared	Predicted R-Squared	Adequate Precision
0.9789	0.9614	0.7907	21.926

**Table 6:** The ANOVA table for the average WSD of the MoS_2_ nanolubricants.

Source	Sum of Squares	Degrees of Freedom (df)	Mean Square	F-Value	*p*-Value Prob > F	Significance
Model	6.339 × 10^−5^	4	1.585 × 10^−5^	31.53	0.0001	Significant
A-Temperature	1.584 × 10^−6^	1	1.584 × 10^−6^	3.15	0.1191	-
B-Time	2.496 × 10^−5^	1	2.496 × 10^−5^	49.66	0.0002	-
AB	1.641 × 10^−5^	1	1.641 × 10^−5^	32.66	0.0007	-
A2	1.955 × 10^−6^	1	1.955 × 10^−6^	3.89	0.0892	-
Residual	3.518 × 10^−6^	7	5.026 × 10^−7^	-	-	-
Lack of Fit	2.466 × 10^−6^	3	8.220 × 10^−7^	3.13	0.1499	Not significant
Pure Error	1.052 × 10^−6^	4	2.630 × 10^−7^	-	-	-
Cor Total	6.691 × 10^−5^	11	-	-	-	-

**Table 7:** The model summary of the quadratic model for the average WSD.

R-Squared	Adjusted R-Squared	Predicted R-Squared	Adequate Precision
0.9474	0.9174	0.7367	20.112

**Table 8:** The model validation for the MoS_2_ nanolubricants.

Response	Predicted	Experimental	% Error
COF	0.0833	0.0849	1.88
Average WSD (μm)	318	320	0.625

**Table 9:** The percentage enhancements of the COF and average WSD after adding MoS_2_ nanoparticles.

Response	Base Oil + MoS_2_ Nanoparticle	Base Oil	% Reduction
COF	0.0849	0.0946	10.25
Average WSD (μm)	320	345	10.60

## Data Availability

The data presented in this study are available on request from the corresponding author.
